# From microbe to metaphor: virus-like problems in organizations

**DOI:** 10.3389/fpsyg.2023.1193895

**Published:** 2023-07-20

**Authors:** Dustin J. Sleesman, Cory E. Cronin

**Affiliations:** ^1^Department of Business Administration, Lerner College of Business and Economics, University of Delaware, Newark, DE, United States; ^2^Appalachian Institute to Advance Health Equity Science, Ohio University, Athens, OH, United States

**Keywords:** problem-solving, virus metaphor, public health, multidisciplinary, organizational leadership, COVID-19

## Abstract

Despite the important role of problem-solving in organizations, our understanding of the fundamental nature of problems is limited. To generate insights and discussion on this topic, we introduce the metaphor of a “virus-like” problem, which is a special kind of problem that often escapes the awareness of organizational leaders. Virus-like problems differ from other problems in organizations because, just like actual viruses, they are hidden, their source is difficult to identify, and they can quickly spread to others. Integrating the public health and organizational psychology fields, we draw lessons from the COVID-19 pandemic and how it was (mis) managed by public officials to offer a new perspective on problems in organizations and offer practical ideas for how leaders can address virus-like problems of their own.

## 1. Introduction

Problem-solving is a crucial aspect of organizational functioning. In fact, an organization’s success is largely dependent on its members’ ability to discover problems and successfully implement solutions to them (Cyert and March, 1963). Most research in this area has focused on how problems can trigger various processes and outcomes. For example, problems often lead to the formation of teams to help facilitate diverse perspectives and creative solutions that would be difficult for individuals to achieve on their own (Hargadon and Bechky, 2006; Shalley and Perry-Smith, 2008). Problems may also create a need for leaders to cognitively reframe challenging situations to help coordinate effective solutions (Reiter-Palmon and Illies, 2004; Sleesman et al., in press). Still other research has found that problems are commonly at the heart of employee disagreements and conflict resolution (Tjosvold et al., 2014; Weitzman and Weitzman, 2014).

Although research has advanced our understanding of problems as antecedents to action, there is a need for more work on the characteristics of problems. To date, the thrust of attention to this topic focuses on differentiating complex and non-complex problems. Three of the most popular constructs in this domain are ill-structured problems (Simon, 1973), adaptive problems (Heifetz et al., 2009), and wicked problems (Rittel and Webber, 1973). There are some nuances among these perspectives. For instance, the ill-structured and adaptive problem frameworks have typically been applied to organizational contexts, whereas wicked problems tend to be studied in policy or large-scale social systems. Further, effective solutions to adaptive and wicked problems typically require behavior change, whereas the ill-structured perspective does not have this emphasis. Despite some minor differences, all three views emphasize features of complexity such as the presence of uncertainty, lack of any obvious or single solution, and disagreement among stakeholders about assumptions or values. Examples of such complex or “messy” problems are an executive team reformulating a corporate strategy in response to competitor actions or a political leader attempting to rebrand their image following lackluster public polling. By contrast, examples of relatively non-complex problems include needing to create an optimal production schedule or implement new communication software for a group of employees.

To stimulate research and offer some practical advice for organizational leaders, we depart from this emphasis on complexity by offering a new perspective that contrasts conventional and “virus-like” problems. Conventional problems are common and include the complex and non-complex examples we just discussed (e.g., reformulating a corporate strategy, creating an optimal production schedule, etc.). By contrast, we assert that some organizational problems are more insidious and often overlooked: those that resemble the characteristics of viruses. As the COVID-19 pandemic reminds us, if they are not managed properly, viruses can spiral out of control and wreak havoc. With this backdrop, we introduce the novel metaphor of “virus-like” problems and describe how they can be managed by organizational leaders.

## 2. Virus-like problems in organizations: visibility, origin identifiability, and transmissibility

Metaphors are a useful tool to help describe new topics, and they can serve as a creative spark that inspires discussion and ideas (Cornelissen, 2006). Scholars have employed numerous metaphors to represent various aspects of organizations. For instance, the “garbage can” view of decision-making considers individuals, problems, and solutions as loosely structured and disorganized aspects of organizations that come together in a chaotic rather than rational manner (Cohen et al., 1972). The escalation of commitment literature uses the metaphor of being “stuck in mud” to depict people who feel entrapped in failing endeavors after investing heavily in them (Sleesman et al., 2012). Negotiation scholars rely on a pie metaphor to represent value, such that when mutually beneficial tactics are used at the bargaining table, negotiators can “expand the pie” and unlock more value for both sides (Fisher et al., 2011).

### 2.1. Learning from the COVID-19 Pandemic

**Figure 1:**
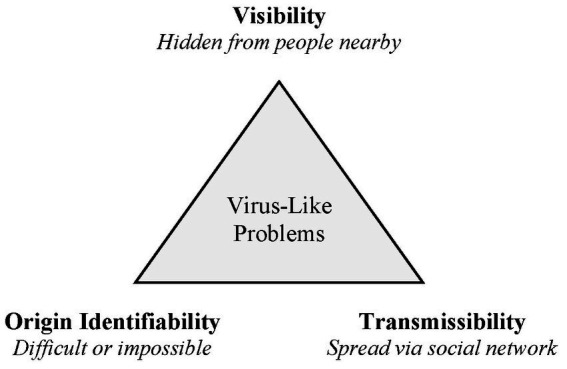
Characteristics of virus-like problems in organizations.

To advance a new way of thinking about organizational problems, we introduce a virus metaphor that describes a special type of problem—one that differs from conventional problems (Nickerson and Zenger, 2004) in three ways: visibility, origin identifiability, and transmissibility (see Figure 1). *Visibility* refers to how obvious or well-known the problem is to people who are associated with it (for example, those in the same department where the problem is occurring). Conventional problems tend to be conspicuous; their existence is self-evident, although there may be some differing opinions about how they should be solved. By contrast, virus-like problems are often hidden or unspoken, even among those who are in close proximity to them. Previous literature on organizational problems does not give much attention to this distinction, although the adaptive problems framework does acknowledge that individuals may sometimes overlook problems (Heifetz et al., 2009).

*Origin identifiability* describes the extent to which the source of the problem can be easily determined. It is usually straightforward to pinpoint how a conventional problem began, but it can be hard or even impossible to track the source of a virus-like problem. Much like the visibility aspect, past research on problems rarely focuses on the trackability of a given problem’s original source. A key exception is the wicked problems view, which maintains that problems can be symptoms of other problems—such as city crime being associated with poverty or lack of opportunity (Rittel and Webber, 1973). However, as noted earlier, wicked problems tend to be associated with policy and social systems rather than organizations, which is our focus.

Lastly, perhaps the most important characteristic, *transmissibility* refers to whether the problem can spread through a social network. Conventional problems may or may not involve social aspects, but a fundamental attribute of a virus-like problem is the potential for social contagion (Ugander et al., 2012). It can exponentially spread from person to person, so ignoring the problem can lead to escalating consequences (Sleesman et al., 2018). This is a unique aspect of the virus-like problem metaphor, as current theoretical understanding of organizational problems does not explicitly account for such contagion effects. In short, a virus-like problem is hidden from many people nearby, has a source that is difficult or impossible to identify, and can spread through a social network.

Table 1 describes some examples of attitudes and behaviors that can manifest as virus-like problems in organizations. To illustrate, suppose an employee has ongoing spats with a manager, and his frustration and bitterness has turned into a sense of cynicism. He shares his discontent with a few colleagues, and over time, they start to sympathize with his concerns. In turn, they develop their own cynical attitudes, which begin to shape their conversations with others, and so on. The cynicism has promulgated throughout the organization to “infect” others many times over (Li and Chen, 2018). In fact, the earlier-mentioned employee may not even be the original source of the cynicism, as he could have been negatively affected by yet another employee’s negative attitude beforehand. As another example, suppose a senior administrator often engages in passive aggressive behavior toward her direct reports. She quietly sabotages their work to hurt their chances for promotion and gives them unnecessary tasks with tight deadlines as indirect punishment for disagreeing with her. As it turns out, she had learned these abusive behaviors from a past manager, who learned it from a different one. It eventually became a learned belief about how managers should treat employees (Li et al., 2020).

**Table 1:** Examples of virus-like problems in organizations.

Attitude or behavior	Example
Cynicism	An employee’s cynicism of management is privately shared with colleagues, who are then primed to notice additional concerns, triggering their own cynical attitudes. These “infected” employees share their cynicism *via* interactions with other colleagues, etc.
Abusive supervision	A senior administrator engages in passive aggressive behavior toward her followers. This eventually results in an unspoken belief about how managers should treat employees, as other managers begin to adopt passive aggressive actions toward their employees, etc.
Stress and burnout	During lunch with a high-achieving coworker, a young employee discovers that he has been quietly tolerating severe work-related stress and burnout for several years. Shaped by this interaction, the young employee learns to overlook his own mental health concerns at work, and he later passes this expectation to future employees, etc.
Work withdrawal	An employee is increasingly disengaged from work, putting in fewer hours, taking longer breaks, and doing the bare minimum requirements for the job (sometimes called “quiet quitting”; Harter (2022). A colleague notices these behaviors, and he – either consciously or subconsciously – adjusts his behavior to become similarly withdrawn from work. In turn, another colleague starts to notice this change, etc.

Just like the cynicism problem, the abusive supervision problem is essentially hidden from many people in the organization. Except for those directly involved, even employees in the same area may not even know about it. The organization operates “business as usual” even though the affected (or infected) people may silently suffer the consequences (O'Donovan et al., 2021). In addition, the origins of these problems are not easily identifiable. They may have even originated outside the organization, brought in vis-à-vis the attitudes or behaviors of a new hire—akin to a “viral hitchhiker” in molecular biology (Cole et al., 2005). Lastly, the problems are transmissible, as they replicated from person to person over time, taking on a life of their own—even becoming normalized. As these characteristics demonstrate, virus-like problems can be especially challenging.

To better understand how leaders can manage such problems that resemble viruses, we distill some critical lessons from how the SARS-CoV-2 virus was managed (or mismanaged) during the COVID-19 pandemic. Drawing from retrospective analyses of how the pandemic unfolded and play-by-play accounts by key decision-makers (Gottlieb, 2021; Lewis, 2021; Slavitt, 2021), our article describes three primary mistakes that resulted in public officials mismanaging the pandemic: lack of problem ownership, insufficient testing, and poor implementation of interventions. By looking back at the mistakes that were made, we hope to guide leaders in avoiding these same mistakes as they confront their own virus-like problems.

### 2.2. Mistake #1: lack of problem ownership

As the SARS-CoV-2 virus began to spread in early 2020, government officials throughout the world were slow to act. This was especially pronounced in the United States whereby no single agency or person took ownership of the problem (Gottlieb, 2021). To worsen matters, messaging from the White House continued to downplay the severity of the pandemic even as it grew worse over time (Summers, 2020). There was no clear call to action. State governors assumed that the federal government would take charge, but they were pushing responsibility back down to the states—leaving no one in control as the pandemic accelerated.

The consequence of this lack of problem ownership was a delay in the ability to confront the pandemic and growing public health issue head-on. For example, the development of COVID-19 testing kits was largely decentralized. Commercial labs and test manufacturers did not coordinate efficiently, and some of the kits being created would only work in certain labs or on instruments that were not widely used (Gottlieb, 2021). The development and clinical trials of COVID-19 treatments faced similar issues. Drug manufacturers and laboratory facilities had to step up on their own to advance treatment research, which resulted in a duplication of work and missed opportunities for collaboration and efficiencies. However, there were some exceptions. For example, the United Kingdom rolled out a streamlined national trial platform called RECOVERY (Randomized Evaluation of COVID-19 Therapy) that was very effective. It was simple, efficient, and well-coordinated among stakeholders, serving as a prominent example of how clinical trials can benefit from integrated efforts (Park et al., 2021).

From a leadership perspective, it is important to recognize the lack of ownership that led to an inability to contain a viral problem. Leaders must be proactive, but this cannot be achieved without recognition of responsibility. For instance, leaders may hear about a virus-like organizational problem (like staff burnout or elevated conflict) and rather than taking time to learn more and help identify the root cause, they may simply delegate its solution to lower-level managers and hope that the issue gets resolved. The reality is that those individuals often have a myopic view of the problem, leading them to seek a localized solution like adding some discussion points to the next department meeting—or delegating the problem even further down the hierarchy by asking supervisors to figure out a solution in their teams. As the problem gets pushed to lower echelons of the organization, it becomes increasingly difficult to address any system-wide issues, such as administrative policies or initiatives set by senior leaders. Further, the longer that no one (particularly high-level leadership) takes ownership of the problem, the more it is likely to multiply in scale and severity.

### 2.3. Mistake #2: insufficient testing

During the early stages of the COVID-19 pandemic, we did not have a diagnostic test and thus could not effectively track the spread of the virus or learn about its symptoms or other characteristics. Given this lack of robust diagnostic testing, health officials had to rely on syndromic surveillance, which refers to collecting and analyzing different streams of information to monitor the prevalence of disease—such as keeping track of how many people visit emergency rooms for respiratory symptoms or logging increases in medicine purchases. Even when testing became more readily available, appropriate usage of them was another challenge. Altogether, these delays and inconsistencies in testing and tracking the virus led to greater spread and increased risk, much to the frustration of public officials.

Although the public health community is well-versed in the importance of testing for diseases, the same *cannot* be said about most leaders in organizations who face virus-like problems in their ranks. Problem detection is often limited to casual observations or hallway conversations (e.g., “I heard that Henry’s team is having lots of conflict these days”). Although it may seem difficult to reliably measure virus-like problems like the ones that appear in Table 1, organizational scientists have developed measures for them that can be accurately assessed using confidential surveys (Church and Waclawski, 2017). This can help to disentangle symptoms of problems (e.g., absenteeism or a lack of communication) from their root causes (like a growing perception of discrimination or low job satisfaction).

Looking back at the COVID-19 pandemic reminds us that such diagnostic testing is only one form of problem detection. To address virus-like problems, leaders should also borrow from the public health community’s notion of syndromic surveillance testing, since it has a broader scope of detection that can reveal trends and patterns over time. Examples include formalizing exit interviews of employees who are departing the organization or institutionalizing a well-designed annual survey that captures perceptions and feedback among various members of the organization. Such a comprehensive, data-driven approach to testing allows leaders to detect emerging problems early and discover clues about how to proactively put interventions into place before they become a serious problem. This process is becoming easier and more effective with recent advancements in data analytics and artificial intelligence (Raisch and Krakowski, 2021).

### 2.4. Mistake #3: poor implementation of interventions

A public health best practice is to ensure an intimate relationship between testing and interventions, and this need was made very clear during the pandemic. Before effective COVID-19 tests were developed, officials in many countries enacted sweeping interventions to “slow the spread” by controlling the rapid growth of infections and hospitalizations, including stay-at-home orders and the temporary closure of businesses and schools. After some time, these measures were replaced with others, including masking, physical distancing, and contract tracing. Officials frequently changed their recommendations, which were sometimes contradictory, like when guidance from the Centers for Disease Control and Prevention (CDC) was incompatible with statements issued by the White House (Summers, 2020). To illustrate, shortly after the CDC recommended mask wearing in April of 2020, President Trump undercut their message, emphasizing that it was merely optional, stating: “So with the masks, it’s going to be, really, a voluntary thing. You can do it. You don’t have to do it. I’m choosing not to do it, but some people may want to do it, and that’s okay.” (The White House, 2020).

Such inconsistencies resulted in a lot of confusion in the public (Scott, 2022), and many people interpreted the public health interventions as capricious, misinformed, or even deceptive (Jamieson, 2021). Messaging was further complicated by the politicization of mitigation efforts as some politicians and their allies sought to limit the authority of public health officials. Partisan legislative bodies even went so far as to enact laws that prevent public officials from initiating mitigation efforts in the future (Weber and Barry-Jester, 2021). The mixed messaging and confusion resulted in more sickness and deaths than there otherwise might have been had interventions been implemented more effectively, and they sowed the seeds of greater distrust in government and public health authorities if a pandemic were to occur again.

The lesson for leaders in organizations is to clearly explain the purpose of any intervention for a virus-like problem. Taking ownership of a problem and identifying its prevalence do little good if they aren’t followed by appropriate interventions that are implemented in clear and effective ways. Fixes (like a policy change or professional development workshop) should be firmly endorsed by leaders, who should plainly articulate how they are tied to organizational goals. It is imperative that leaders also model the desired attitudes or behaviors, particularly given how influential they can be in shaping organizational culture and norms (Metwally et al., 2019). To truly address virus-like problems, interventions must be carefully implemented. Otherwise, as the pandemic has shown, they may not have any positive effects—and they may even backfire by making the problem worse.

## 3. Discussion

We encourage leaders to meet the needs of their organization as they would a customer, investor, or any other key stakeholder—and this often involves managing virus-like problems. Just like an actual virus, this special kind of problem is hidden to many people, it is difficult to track its source, and it may quickly spread to others. These characteristics make virus-like problems especially pernicious, even compared to complexity-oriented problems that have been identified in past literature (e.g., ill-structured, adaptive, and wicked problems). To be clear, complex problems may be very challenging and important, but the stealthy, exponential spread of virus-like problems makes them a unique and time-sensitive threat that should not be ignored.

As we have highlighted in this article, organizational leaders should focus on three key tactics to successfully address virus-like problems: they should proactively take ownership of such problems, deploy robust testing procedures to understand them, and implement interventions that are clear and meaningful to people. But what else can be done to tackle this special breed of problems in organizations?

First, leaders should establish cultures of accountability and transparency. This involves having candid conversations with their peers and other employees regarding virus-like problems and exchange ideas for how to address them. Genuine discussions of these topics are unlikely to naturally occur given time constraints and power dynamics, so it is the leader’s responsibility to invite open dialogue and honest self-reflection (Newman et al., 2017). Our introduction of a virus metaphor may resonate well with people and serve as a vivid catalyst for discussion, given everyone’s shared experience of the COVID-19 pandemic. By encouraging accountability and transparency, leaders can have greater confidence in their organization’s ability to identify and address tough virus-like problems.

In addition, investing time to build and maintain collaborative relationships within one’s organization can play an important role in dealing with virus-like problems. The human resources (HR) department, for example, should be viewed as a strategic partner to help identify virus-like problems and implement effective interventions (such as developing surveys, workshops, and other solutions that we noted earlier). Too many leaders view HR as merely serving an administrative or clerical function, but they are well-positioned to enact organization-wide initiatives to understand employee concerns, build trust, and establish the kind of work environment that is equipped to handle virus-like problems before they escalate.

As we all witnessed throughout the COVID-19 pandemic, leaders can exacerbate a sense of uncertainty, fear, or distrust within a culture—but they also have the power to make things better. By paying more attention to virus-like problems, leaders can increase their capability to ensure that their organization remains healthy. On the academic front, we hope to inspire future research that unpacks the virus metaphor and ultimately advance a better understanding of the nature of problems in today’s inter-connected and dynamic organizations.

## Data availability statement

The original contributions presented in the study are included in the article/Supplementary material, further inquiries can be directed to the corresponding author.

## Author contributions

The article idea was proposed by DS and revised by CC. The first draft of the manuscript was written by DS and revised by CC. All authors contributed to the article and approved the submitted version.

## Conflict of interest

The authors declare that the research was conducted in the absence of any commercial or financial relationships that could be construed as a potential conflict of interest.

## Publisher’s note

All claims expressed in this article are solely those of the authors and do not necessarily represent those of their affiliated organizations, or those of the publisher, the editors and the reviewers. Any product that may be evaluated in this article, or claim that may be made by its manufacturer, is not guaranteed or endorsed by the publisher.
